# Circulating soluble endoglin modifies the inflammatory response in mice

**DOI:** 10.1371/journal.pone.0188204

**Published:** 2017-11-16

**Authors:** Laura Ruiz-Remolina, Claudia Ollauri-Ibáñez, Lucía Pérez-Roque, Elena Núñez-Gómez, Fernando Pérez-Barriocanal, José Miguel López-Novoa, Miguel Pericacho, Alicia Rodríguez-Barbero

**Affiliations:** 1 Department of Physiology and Pharmacology, University of Salamanca, Salamanca, Spain; 2 Biomedical Research Institute of Salamanca (IBSAL), Salamanca, Spain; Centre National de la Recherche Scientifique, FRANCE

## Abstract

Inflammation is associated with every health condition, and is an important component of many pathologies such as cardiovascular diseases. Circulating levels of soluble endoglin have been shown to be higher in the serum of patients with cardiovascular diseases with a significant inflammatory component. The aim of this study was to evaluate the implication of circulating soluble endoglin in the inflammatory response. For this purpose, a transgenic mouse expressing human soluble endoglin (sEng+) was employed, and three different inflammatory approaches were used to mimic inflammatory conditions in different tissues. This study shows that control sEng+ mice have a normal inflammatory state. The lung and kidney injury induced by the inflammatory agents was reduced in sEng+ mice, especially the intra-alveolar and kidney infiltrates, suggesting a possible reduction in inflammation induced by soluble endoglin. To deepen into this possible effect, the leukocyte number in the bronchoalveolar lavage and *air pouch* lavage was evaluated and a significant reduction of neutrophil infiltration in LPS-treated lungs and ischemic kidneys from sEng+ with respect to WT mice was observed. Additionally, the mechanisms through which soluble endoglin prevents inflammation were studied. We found that in sEng+ animals the increment of proinflammatory cytokines, TNFα, IL1β and IL6, induced by the inflammatory stimulus was reduced. Soluble endoglin also prevents the augmented adhesion molecules, ICAM, VCAM and E-selectin induced by the inflammatory stimulus. In addition, vascular permeability increased by inflammatory agents was also reduced by soluble endoglin. These results suggest that soluble endoglin modulates inflammatory-related diseases and open new perspectives leading to the development of novel and targeted approaches for the prevention and treatment of cardiovascular diseases.

## Introduction

Inflammation is the body’s response to tissue injury, infection or invasion by microorganisms and its purpose is to keep maintain homeostasis [1]. Inflammation has been found to be associated with every health condition, and is an important secondary component of many pathologies. Inflammation, often named the *secret killer*, is a key factor in a large group of diseases affecting and killing millions of people every year. Examples of inflammatory diseases or diseases with key inflammatory components include arthritis, cardiovascular diseases, hypertension, obesity, insulin resistance, arthritis, alzheimer’s and parkinson’s diseases, peritonitis, colitis and several types of cancer [2,3]. An impaired immune response is a driver in the pathogenesis of many diseases, and its untimely resolution represents a process, which if targeted, could provide new therapeutic avenues for a multitude of diseases treatments. Currently, anti-inflammatory drugs, both NSAIDs and steroidal, fail to deliver a solution because they tackle only parts of the inflammatory cascade and induce unacceptable side effects. Drug hypersensitivity reactions are increasing in the 21^st^ Century with the ever-expanding availability of new therapeutic agents. During inflammation there is an increased production of various mediators, including proinflammatory cytokines and eicosanoids [4], which have been used as both inflammation biomarkers and therapeutic targets. Soluble endoglin (sEng) is emerging as a new biomarker for several cardiovascular diseases. Circulating levels of soluble endoglin have been shown to be higher in the serum of patients with preeclampsia, hypercholesterolemia, atherosclerosis, diabetes mellitus and hypertension [5–8], diseases which all have a significant inflammatory component [2,3].

Soluble endoglin is a glycoprotein generated by the cleavage of the extracellular domain of the membrane-bound endoglin [5] by a MT1-MMP (membrane-type metalloprotease-1) as the main endoglin-shedding protease [9]. Increased levels of soluble endoglin in plasma have been correlated with predicting complications in myocardial infarction [10], acute heart failure [11,12], cardiovascular events in patients with chronic coronary artery disease [13], cardiovascular alterations in patients with hypertension and diabetes, [8] and has diagnostic and prognostic value in preeclampsia [5]. Soluble endoglin appears to be a good biomarker of damage in diseases in which it has been related. However, how endoglin is involved in the pathogenesis has not been researched in-depth. There are some studies postulating that the effect of soluble endoglin is produced by antagonizing the effect of membrane endoglin or by sequestering the ligand. Soluble endoglin can bind several ligands, among them TGF-β1, BMP-9 and BMP-10 [5,14]. When soluble endoglin binds circulating TGF-β1, the availability of this cytokine to interact with its membrane receptors decreases, as soluble endoglin is unable to interact directly with the extracellular region of TGF-β receptors type I and type II [15].

To gain a wide-ranging understanding of the therapeutic areas of inflammatory diseases, we have study the effect of soluble endoglin on inflammation. The implication of soluble endoglin on the inflammation related-diseases could open a new therapeutic area for the treatment of these diseases. The aim of this study was to evaluate the implication of soluble endoglin in the inflammatory response of an *in vivo* animal model. To this end, a transgenic mouse model expressing human soluble endoglin (sEng+) was employed, and three different inflammatory approaches, lipopolysaccharide in lung, carrageenan in air pouch and renal ischemia-reperfusion, were used to mimic the inflammatory conditions. This model permits the direct relation between circulating soluble endoglin and the inflammatory processes to be analyzed.

## Materials and methods

### Ethics statement

All animal procedures were conducted in strict compliance with the European Community Council Directive (63/2010/UE) and Spanish legislation and the protocols were approved by the University of Salamanca Ethics Committee. The animals were housed under SPF conditions at the SEA Animal House of the NUCLEUS platform at the University of Salamanca (ES372740000046).

### Reagents

λ-Carrageenan (catalog #2329535), lipopolysaccharide (LPS) *(*catalog #L3129) and Fluorescein Isotiocyanate Dextran (FITC-Dextran) (catalog #FD40) were purchased from Sigma-Aldrich (St Louis, MO, USA). Recombinant human endoglin (rhEndoglin; Eng) (Catalog #1097-EN-025) was purchased from R&D Systems (Minneapolis, MN, USA). Isoflurane and buprenorphine (Schering-Plough; Madrid, Spain) were used for anesthesia and analgesia respectively.

### Mice

A mouse line expressing human soluble endoglin (sEng+) was generated at the Genetically Modified Organism Unit (NUCLEUS Platform, University of Salamanca, Spain) as it has been already reported [16]. The cDNA corresponding to the extracellular domain of endoglin was cloned under the control of the CMV-actin gene promoter. Linear DNA fragments for microinjection were obtained by SalI/KpnI digestion and injected into CBA x C57BL/6J fertilized eggs as previously described [16]. The progeny was screened for the endoglin transgene by polymerase chain reaction analysis of tail DNA. The studies reported here were performed on the F7 generation of 6-8-month-old male mice, weighing 25–30 g. The animals were given standard laboratory chow (Panlab; Barcelona, Spain) and water ad libitum, and housed in the animal experimental service at NUCLEUS Platform, with a temperature of 22–24°C, humidity of 60–65%, and 12 h light/dark cycles, along with their wild-type littermates. Animals showed no signs of severe illness following any of the laboratory procedures. After surgery, a single dose of the analgesic buprenorphine was injected subcutaneously to reduce pain in the post-surgery phase. Animals were kept warm during 3 h, and the condition of the animals was monitored every 3 h after the laboratory procedure. No animals died due to the experimental procedures. Mice were always euthanized in a CO_2_ chamber.

### Study design

#### Lipopolysaccharide-induced acute lung injury

**Fig 1:**
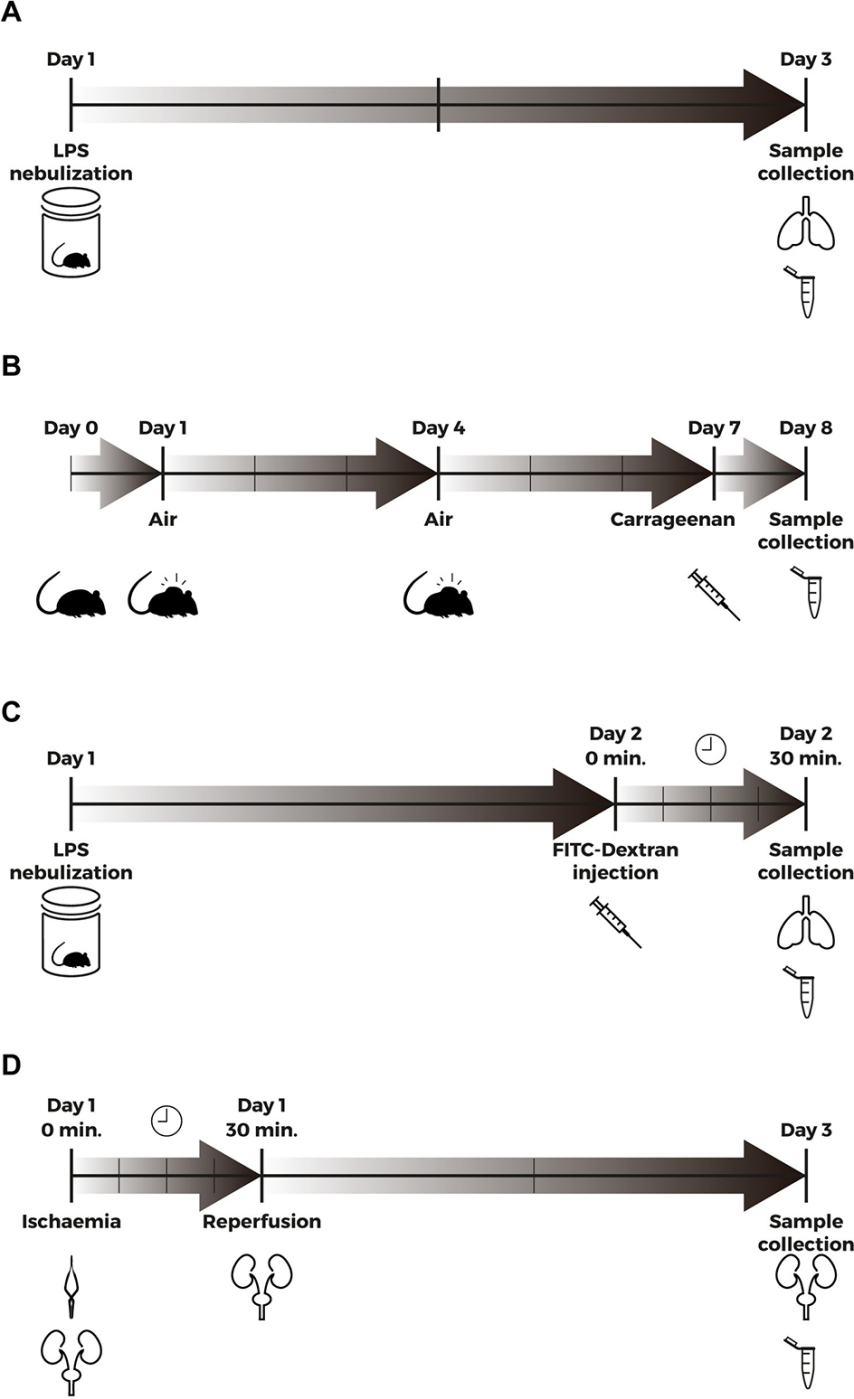
Methods timeline. (A) Aerosolized LPS (5mg/ml) was administered by inhalation to WT and sEng+ mice (Day 1). 48 h after LPS exposure, mice were euthanized and BAL was carried out by instilling sterile PBS into the lungs via tracheotomy (Day 3). BAL was used for the analysis of proinflammatory cytokines, leukocytes and protein determination. (B) An air pouch was created on the back of WT and sEng+ mice by subcutaneous injection of 3 ml filtered air (Day 1). The pouch was re-inflated with an additional 2 ml of filtered air (Day 4). Inflammation was induced by injecting 500 μl of 1% carrageenan in PBS into the air pouch of the anesthetized mice (Day 7). Mice were sacrificed and air pouch lavages were performed with 2 ml of PBS (Day 8). Air pouch lavage was used for analysis of proinflammatory cytokines, leukocytes and protein determination. (C) Aerosolized LPS (5mg/ml) was administered by inhalation to WT and sEng+ mice (Day 1). 24 h after LPS exposure, 40 kDa FITC-Dextran (25 mg/ml) was injected into the retroorbital venous sinus to WT and sEng+ mice. 30 min later, the mice were euthanized (Day 2), and BAL was collected by instilling PBS into the lungs via tracheotomy. (D) Male mice were anaesthetized by isoflurane inhalation. Following abdominal incision, left renal pedicle was bluntly dissected and a microvascular clamp was placed on the left renal pedicle for 30 min. After a 30-min ischemia, the clamps were removed and the wounds sutured. After closure, animals were subcutaneously injected with 0,5 ml of PBS (Day 1). 48 h later, the mice were euthanized and blood and the kidneys were collected (Day 3).

The study was carried out using a mouse model of acute lung injury (ALI) induced by aerosolized administration of LPS (Fig 1A). Mice were randomly divided into two groups: the sham group (control), which were exposed to nebulized NaCl solution, and the LPS group (LPS), which were exposed to nebulized LPS. The mice were placed into a chamber and exposed to aerosolized LPS (5 mg/ml in sterile 0,9% NaCl solution) for 30 min using a nebulizer unit driven by a pressure of 2 bars. The control, vehicle treated mice, were aerosolized with a 0,9% NaCl solution. 48 h after LPS exposure, the animals were euthanized and blood was collected through cardiac puncture and later centrifuged at 7 000 g for 4 min at 4°C for obtaining a plasma sample. Then, bronchoalveolar lavage (BAL) was carried out by instilling sterile endotoxin-free phosphate buffered saline (PBS) into the lungs via tracheotomy, which was then centrifuged at 300 g for 10 min at 4°C. The supernatant was subsequently collected and analyzed for the presence of proinflammatory cytokines, and protein levels were determined. The cell pellet was resuspended in PBS and used for leukocyte quantification using a Neubauer hemocytometer. The protocol is available in http://dx.doi.org/10.17504/protocols.io.j8ecrte.

The lungs of each animal were removed and divided into two parts: the left lower lobe was snap-frozen and later processed for obtaining lung homogenates, and the rest of the material was fixed in 4% formalin for histological evaluation.

#### Carrageenan-induced air-pouch inflammation

Air pouches were created on the backs of WT and sEng+ mice by subcutaneous injection of 3 ml of filtered air. The pouches were re-inflated on day 4 with an additional 2 ml of filtered air, and after 7 days a synovial-like epithelium was present in the air pouch [17]. Inflammation was induced by injecting 500 μl of 1% carrageenan in PBS into the air pouch of the anesthetized mice. The pouches of the control mice were injected with only 500 μl of PBS solution. After 24 h, the mice were euthanized, air pouch lavages were performed with 2 ml of PBS (Fig 1B) and the liquid was centrifuged at 300 g for 10 min at 4°C. Air pouch lavages were analyzed for the presence of proinflammatory cytokines, and protein levels were determined. Leukocyte identification was performed using an automated hematology analyzer (ADVIA® 120; Siemens Healthcare, Erlanger, Germany). The protocol is available in http://dx.doi.org/10.17504/protocols.io.j8dcrs6.

#### Ischemia-reperfusion-induced acute kidney injury

Ischemia-reperfusion injury (IRI) is the main cause of acute renal failure and is characterized by an inflammatory reaction, tubular necrosis and fibrosis. In this work, an established model of renal IRI in mice was used [18], where male mice were anaesthetized by isoflurane inhalation. A microvascular clamp (Roboz Surgical Instrument, Rockville, MD, USA) was placed on the left renal pedicle, and after 30 min of ischemia, the clamp was removed and the wound sutured. Then, the animals were left to recover, with free access to food and water. Sham-operated mice underwent the same surgical procedure without clamping the renal pedicle (Fig 1D). 48 h after ischemia, the mice were euthanized, blood was collected through cardiac puncture and later centrifuged to obtain a plasma sample.

The kidneys were removed from each animal and divided into two parts: one was snap-frozen and later processed to obtain kidney homogenates, and the rest was fixed in 4% formalin for histological evaluation. The protocol is available in http://dx.doi.org/10.17504/protocols.io.j8gcrtw.

### Histological analysis

Lung and kidney tissue was fixed in 4% neutral-buffered formalin and subsequently embedded in paraffin. Three-mm thick sections were stained with hematoxylin and eosin (H&E) using a standard protocol [19]. The sections were analyzed by light microscopy and images were taken with an Olympus DP70 digital camera system in an Olympus BX51F microscope. Histological changes were evaluated by a pathologist blind to the treatment regimen. A scoring system to grade the degree of lung and kidney injury was employed [20,21] based on the histological features specified in Tables 1 and 2. Each feature was graded as absent, mild, moderate, or severe, with a score of 0–3. A total score was calculated for each animal.

**Table 1:** Lung injury histopathology score system.

Score	0	1	2	3
**Alveolar septae**	All septae are thin and delicate	Congested alveolar septae in less than 1/3 of the field	Congested alveolar septae in 1/3 to 2/3 of the field	Congested alveolar septae in greater than 2/3 of the field
**Alveolar hemorrhage**	No hemorrhage	At least 5 erythrocytes per alveolus in 1 to 5 alveoli	At least 5 erythrocytes per alveolus in 5 to 10 alveoli	At least 5 erythrocytes per alveolus in more than 10 alveoli
**Intra-alveolar fibrin**	No intra-alveolar fibrin	Fibrin strands in less than 1/3 of the field	Fibrin strands in 1/3 to 2/3 of the field	Fibrin strands in greater than 2/3 of the field
**Intra-alveolar infiltrates**	Less than 5 intra-alveolar cells per field	5 to 10 intra-alveolar cells per field	10 to 20 intra-alveolar cells per field	More than 20 intra-alveolar cells per field

A scoring system to grade the degree of lung injury was employed based on the following histologic features: alveolar septae, alveolar hemorrhage, intra-alveolar fibrin and intra-alveolar infiltration per field. Each feature was graded as absent, mild, moderate, or severe, with a score of 0–3. A total score was calculated for each animal.

**Table 2:** Kidney injury histopathology score system.

Score	0	1	2	3
**Glomerulus**	No damage	Thickening of Bowman capsule	Retraction of glomerular tuft	Glomerular fibrosis
**Tubules**	No damage	Loss of brush border and/or tubular debris	Partial tubular obstruction	Tubular obstruction and dilatation
**Neutrophil infiltration**	No damage	Slight neutrophils surrounding tubules	Moderate neutrophils surrounding tubules	Severe confluent neutrophils surrounding tubules

A scoring system to grade the degree of kidney injury was employed based on the following histologic features: glomerular fibrosis, tubular obstruction and dilation and neutrophil infiltration per field. Each feature was graded as absent, mild, moderate, or severe, with a score of 0–3. A total score was calculated for each animal.

### Protein determination

Protein concentration in BAL and the air pouch lavage was quantified using the DC protein Assay kit (Bio-Rad; Hercules, CA, USA). Absorbance was measured at 750 nm by an absorbance plate reader (BIOTEK Instrument; Winooski, VT, USA) and the results were analyzed using the Gen 5 program (BIOTEK Instrument; Winooski, VT, USA).

### Lung wet-to-dry weight ratio

The lung wet-to-dry weight ratio (W/D) was calculated to assess lung tissue edema. The diaphragmatic lobe of the left lung was excised separately and rapidly weighed to obtain the wet-weight. To determine the stable dry lung weight, the samples were oven dried (60°C) for 48 h.

### Evaluation of lung permeability by FITC-Dextran intravenous injection

FITC-Dextran intravenous injection is a well-established method for measuring lung vascular permeability. 24 h after LPS exposure, 100 μl of 40 kDa FITC-Dextran was injected into the retroorbital venous sinus in WT and sEng+ mice, after 30 min the mice were euthanized. BAL was collected by instilling PBS into the lungs via tracheotomy and the liquid was centrifuged at 300 g for 10 min (Fig 1C). Blood was collected and centrifuged at 7 000 g for 4 min to obtain a plasma sample. The fluorescence intensity (FI) of the FITC-Dextran in BAL and plasma was determined at an excitation wavelength of 485 nm and an emission wavelength of 528 nm [22] using a Synergy H1 plate reader (BIOTEK Instrument; Winooski, VT, USABioTek). The ratio BAL FI/Plasma FI was calculated to assess lung vascular permeability. The protocol is available in http://dx.doi.org/10.17504/protocols.io.j8hcrt6.

### ELISA kits

Plasma levels of human endoglin were determined by means of Human Endoglin/CD105 Quantikine ELISA kit (R&D System; Minneapolis, MN, USA) according to the manufacturer’s instructions.

Myeloperoxidase (MPO) activity in snap frozen kidneys was determined using a MPO Assay Kit (HK210 Hycult®Biotech, Plymouth Meeting, PA, USA) according to the manufacturer’s instructions. All the results were normalized using the tissue weight.

Cytokine levels (TNFα, IL1β and IL6) in tissue homogenates, BAL, air pouch lavage and plasma were measured using the murine cytokine-specific Quantikine ELISA kits (R&D Systems) according to the manufacturer’s protocol. The tissue homogenates were normalized using the tissue weight (mg of tissue per sample).

### Real-time PCR quantification

For RNA extraction, the tissues were ground and the total RNA was isolated using NucleoSpin® RNA columns (Macherey-Nagel; Düren, Germany) according to the manufacturer’s instructions. RNA was quantified using NanoDrop® spectrophotometer ND-1000 (Thermo Scientific Fisher; Waltham, MA, USA). Total RNA was reverse-transcribed into cDNA with a High Capacity Reverse-Transcription kit (iScript^TM^ cDNA synthesis kit; Bio-Rad, Hercules, CA, USA). Real-time PCR detection was performed with a SYBR Green supermix (Bio-Rad, Hercules, CA, USA). Gene specific primers were designed and checked using the BLAST algorithm [23]. Mouse gene expression was normalized using the expression of GAPDH, β-Actin and RPS13. The primer pairs used in the analysis are specified in Table 3. The protocol is available in http://dx.doi.org/10.17504/protocols.io.j8acrse

**Table 3:** Real-time PCR primer pairs.

Mice primers	Forward (5’-3’)	Reverse (5’-3’)
**GAPDH**	GTC GGT GTG AAC GGA TTT G	GAA TTT GCC GTG AGT GGA GT
**β-Actin**	TCT ACA AAT GTG GCT GAG GAC T	GAGGGACTTCCTGTAACCACTT
**RPS13**	GATGCTAAATTCCGCCTGAT	TAGAGCAGAGGCTGTGGATG
**IL1 β**	GCCTGTGTTTTCCTCCTTGC	TGCTGCCTAATGTCCCCTTG
**IL6**	TCCAGTTGCCTTCTTGGGAC	AGTCTCCTCTCCGGACTTGT
**E-selectin**	ATGTGATGATGTCCCTCGGC	CTTCCTGGGTCCACTTTCCC
**VCAM-1**	GAACCCAAACAGAGGCAGAG	GGTATCCCATCACTTGAGCAG
**VE-Cadherin**	ATTGGCCTGTGTTTTCGCAC	CACAGTGGGGTCATCTGCAT

### Western blot assay

Lung and kidney tissues were homogenized in lysis buffer (Tris-ClH pH = 7,5 20mM, NaCl 140mM, EDTA pH = 8 10mM, 10% Glicerol, Igepal CA-630, H_2_O mQ) containing proteinase and phosphatase inhibitors (Roche Diagnostics; Mannheim, Germany). Western blot analysis was performed as we previously described [19]. The primary antibodies used are specified in Table 4. The protocol is available in http://dx.doi.org/10.17504/protocols.io.j8bcrsn.

**Table 4:** Western blot primary antibodies.

Antigen	Supplier	Catalog number	Host species	Dilution
**Endoglin**	MJ7/18[24]		Rat	1:2
**ICAM-1**	R&D Systems	AF796	Goat	1:1 000
**VCAM-1**	Santa Cruz Biotechnology	Sc-1504	Goat	1:1 000
**Calnexin**	Stressgen	SPA-860	Rabbit	1:10 000

### Endothelial paracellular permeability assay

The human umbilical vein endothelial cell line EA.hy926 (ATCC; Mannasas, VA, USA) are hybrid endothelial cells derived by fusing human umbilical vein endothelial cells with the permanent human cell line A549. EA.hy926 were cultured in Dulbecco's Modified Eagle Medium (DMEM) supplemented with 10% fetal calf serum (FCS) in an atmosphere of 5% CO2/95% air and 37°C.

EA.hy926 cells (10^5^cells/plate) were seeded in transwell inserts (Corning® Biocoat^TM^ Cell Culture Inserts Collagen, Type I Rat Tail, 24-Well, 3 μm; Corning, NY, USA) and were cultured to confluence at 24 h. The cells were starved overnight and then activated with LPS (1 μg/ml) for 4 h. Upper chamber media, containing LPS (1 μg/ml) and soluble endoglin (500 ng/ml) for their respective treatments, were replaced with FITC-Dextran (40 kDa) at 1 mg/ml in DMEM. The bottom chambers were also replaced with DMEM. After 24 h at 37°C the inserts were removed, and the amount of fluorescence in the bottom chambers was measured using a fluorescence plate reader (Fluoroskan Ascent FL; Thermo Electron Corporation, Waltham, MA, USA). The protocol is available in http://dx.doi.org/10.17504/protocols.io.j8ccrsw.

### Statistical analysis

The statistical analysis was performed using the GraphPad Prism 6.0 software (GraphPad Software, Inc., CA, USA). All data are expressed as mean ± standard error of mean (SEM). The differences between groups were examined for statistical significance using two-way *ANOVA* with Bonferroni post-hoc analysis or the Student’s *t*-test. *P* values less than 0,05 were considered statistically significant.

## Results

### Soluble endoglin did not modify the membrane endoglin expression

**Fig 2:**
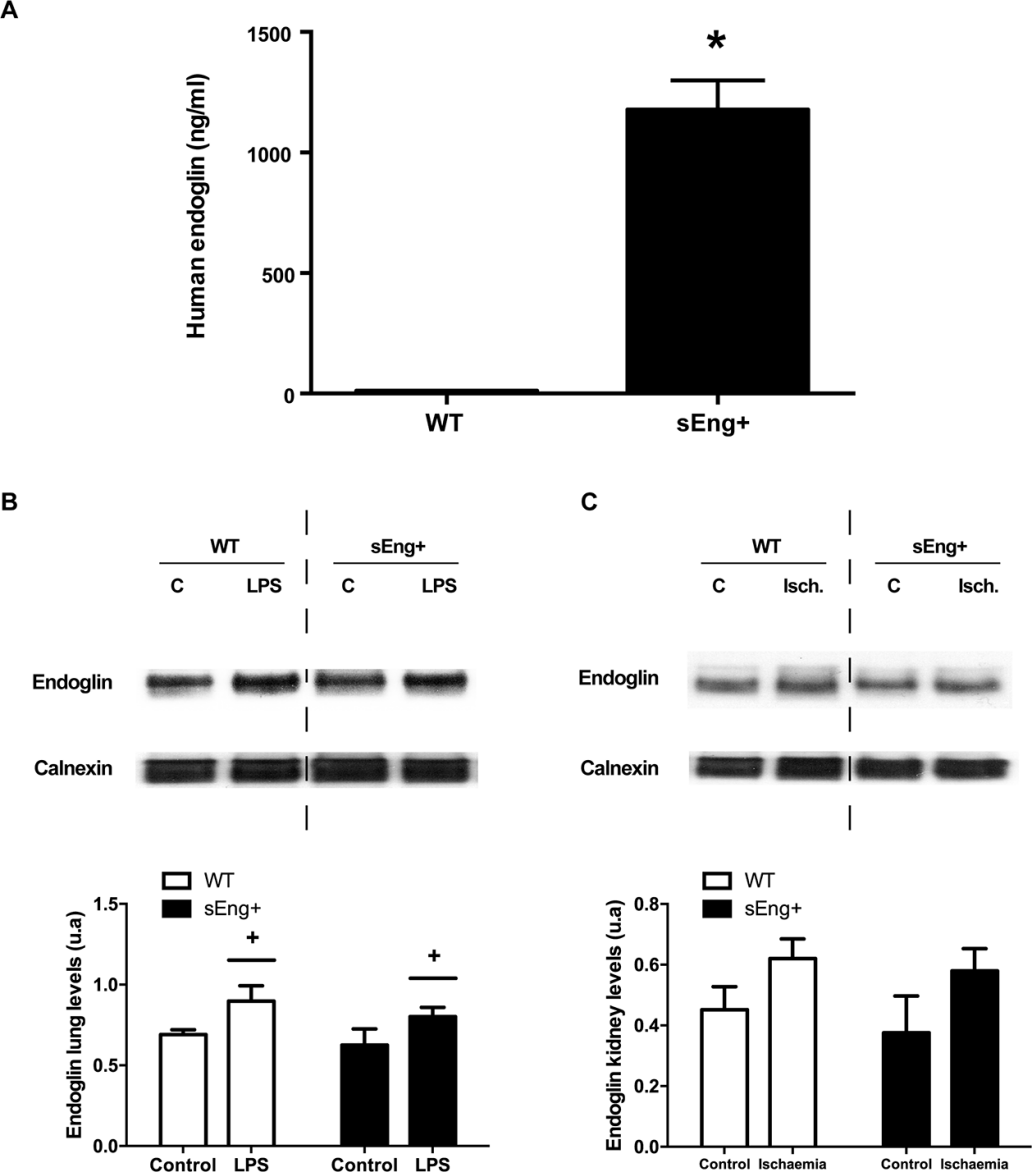
Soluble human endoglin and membrane mouse endoglin in WT and sEng+ mice. (A) Soluble human endoglin was measured by ELISA from plasma of WT and sEng+ mice. Data are expressed as mean ± SEM. n = 20 in each group of mice. *p<0,001, T test. (B) Mouse membrane endoglin amount of protein in the lung was determined by western blot: +p<0,05 LPS *vs* control, two-way ANOVA. (C) Mouse membrane endoglin amount of protein in the kidney was determined by western blot. Equal loading of samples was confirmed by immunodetection of calnexin. Top: Representative immunoblots. Bottom: densitometric analysis. Data are expressed as mean ± SEM. n = 5 in each group of mice.

To corroborate the animal model of human endoglin expression, we determined the concentration of soluble human endoglin in mice plasma. The ELISA analysis showed elevated levels of human endoglin in sEng+ mice and no soluble human endoglin in WT mice (Fig 2A).

We analyzed the amount of membrane endoglin protein in lung and kidney from control and treated WT and sEng+ mice. We observed an increase in the amount of mouse membrane endoglin protein after inflammation in lung and kidney tissues. However, no significant differences between sEng+ and WT mice were found (Fig 2B and 2C).

### Soluble endoglin modified the histopathological changes induced by inflammation in lung and kidney

**Fig 3:**
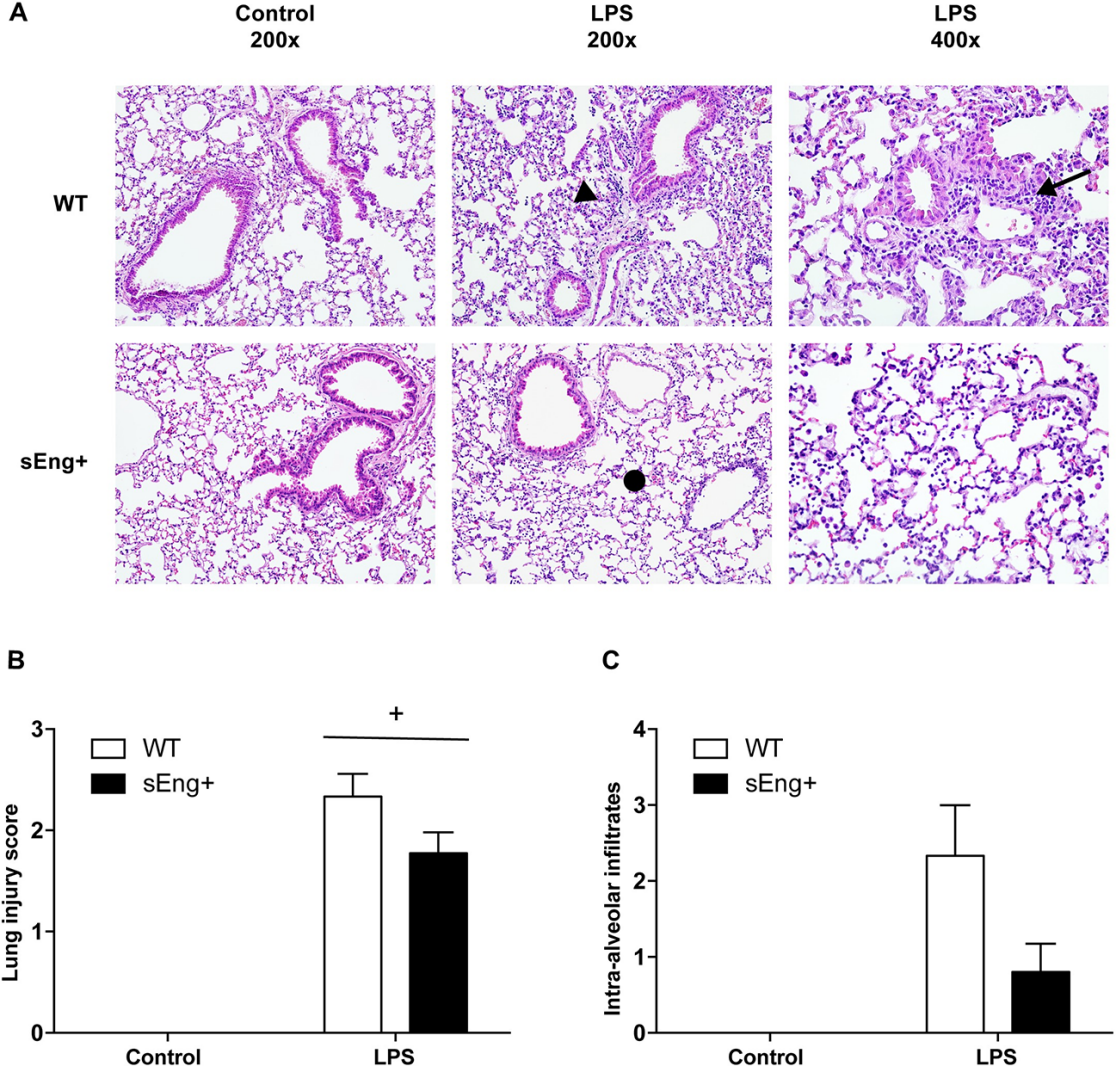
Morphological lung changes after LPS treatment. (A) Representative images of hematoxylin and eosin stained lung sections of five animals from each experimental group. Lungs were fixed with 4% paraformaldehyde, embedded in paraffin, and cut into 5 μm thick sections before being stained. Photomicrographs were obtained with a Nikon Eclipse E800 microscope. Both WT and sEng+ mice lungs show marked inflammatory infiltrates (arrow) after LPS treatment, inter-alveolar septal thickening (arrow head), and interstitial edema (•). Magnification x200 and x400. (B) Severity of lung injury was scored by a pathologist using a semiquantitative histopathology score system which evaluates lung injury in four categories: alveolar septae, alveolar hemorrhage, intra-alveolar fibrin, and intra-alveolar infiltrates. Data are expressed as mean ± SEM. n = 5 in each group of mice, +p<0,0001 *vs* control, two-way ANOVA. (C) Evaluation score of intra-alveolar infiltrates. n = 5 in each group of mice.

ALI is a life-threatening, diffuse heterogeneous lung injury characterized by acute onset, pulmonary edema and respiratory failure. The main features of experimental ALI include at least three out of the following four features: histological evidence of tissue injury, such as the accumulation of neutrophils in the alveolar or the interstitial space; alteration of the alveolar capillary barrier, and as a consequence an increase in the total protein concentration of BAL; an inflammatory response, such as an increase in the absolute number of neutrophils in the BAL; and evidence of physiological dysfunction [25]. To evaluate the potential role of soluble endoglin in this process, histopathological analysis of LPS-induced ALI was performed. H&E staining of lung sections showed normal lung parenchyma and fewer macrophages in the alveolar space of control WT and sEng+ mice, with no significant differences. In addition, indications of marked inflammatory infiltrates, inter-alveolar septal thickening, and interstitial edema in both WT and sEng+ mice lungs after LPS treatment were found (Fig 3A). Interestingly, the patchy areas of neutrophilic infiltration into the lungs were lighter in sEng+ than in WT mice (Fig 3A). Furthermore, severity of lung injury was also scored by a pathologist in a blind fashion, using a semiquantitative histopathology score system already described [25], which classifies lung injury into four categories: alveolar septae, alveolar hemorrhage, intra-alveolar fibrin, and intra-alveolar infiltrates, as show the Table 1. Although soluble endoglin tended to reduce the lung injury (Fig 3B) and intra-alveolar infiltrates score (Fig 3C), the observed differences did not reach statistical significance.

**Fig 4:**
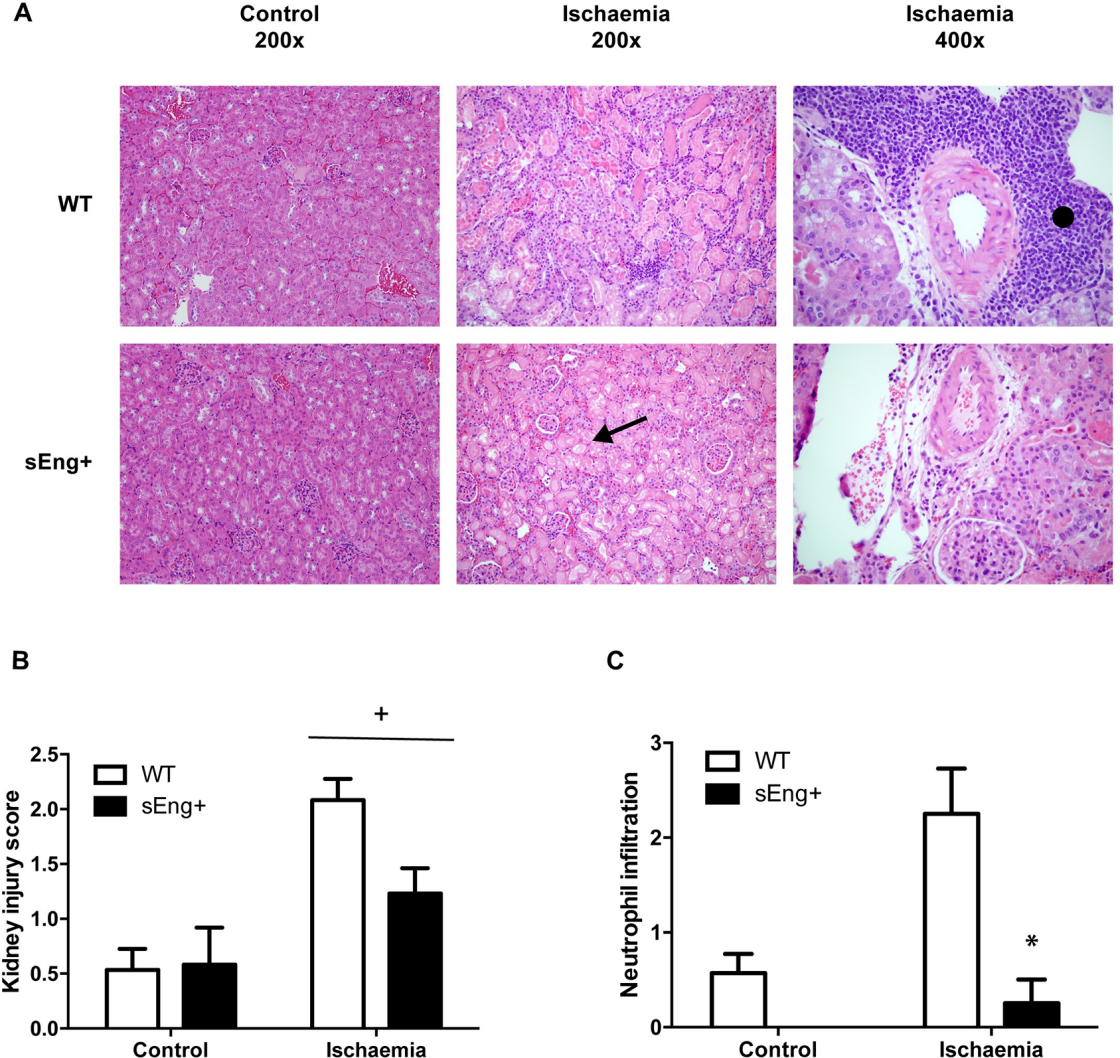
Morphological kidney changes after ischemia-reperfusion. (A) Representative images of hematoxylin and eosin-stained kidney sections from five animals in each experimental group. Kidneys were fixed with 4% paraformaldehyde, embedded in paraffin, and then cut into 5 μm thick sections before being stained. Photomicrographs were obtained with a Nikon Eclipse E800 microscope. Both WT and sEng+ mice kidneys show cortical and medullary hyperemia with areas of tubular necrosis found in the deep and superficial cortex, tubular cast and a significant expansion of the tubular structure with destruction of the epithelium (arrow) and inflammatory infiltrates (•). Magnification x200 and x400. (B) Severity of kidney injury was scored by a pathologist using a semiquantitative histopathology score system which evaluates kidney injury in three categories: glomerular fibrosis, tubular obstruction and dilation and neutrophil infiltration. Data are expressed as mean ± SEM. n = 5 in each group of mice, +p<0,0001 *vs* control, two-way ANOVA. (C) Evaluation score of neutrophil infiltration. n = 5 in each group of mice, *p<0,01 *vs* ischemic WT, T test.

Fig 4 shows H&E staining of kidney sections 48 h after ischemia-reperfusion. Normal renal structure in the control WT and sEng+ mice was observed, without significant differences. Also, indications of cortical and medullary hyperemia with areas of tubular necrosis in the deep and superficial cortex, tubular cast and a significant expansion of the tubular structure with destruction of the epithelium and inflammatory infiltrates were found in both WT and sEng+ mice kidneys after ischemia-reperfusion (Fig 4A). Interestingly, the patchy areas of neutrophilic infiltration into the kidneys were lighter in sEng+ than in WT mice (Fig 4A). Furthermore, severity of kidney injury was also scored by a pathologist in a blind fashion using a semiquantitative histopathology score system already describe [21], which classifies kidney injury into three categories: glomerular damage, tubular obstruction and dilation and neutrophilic infiltration, as show Table 2. Soluble endoglin induced a significant reduction in the scores representing the level of injury (Fig 4B) and a significant reduction in neutrophil infiltration in ischemic kidneys from sEng+ mice was also observed as compared to the WT mice (Fig 4C).

### Soluble endoglin attenuated leukocyte recruitment

**Fig 5:**
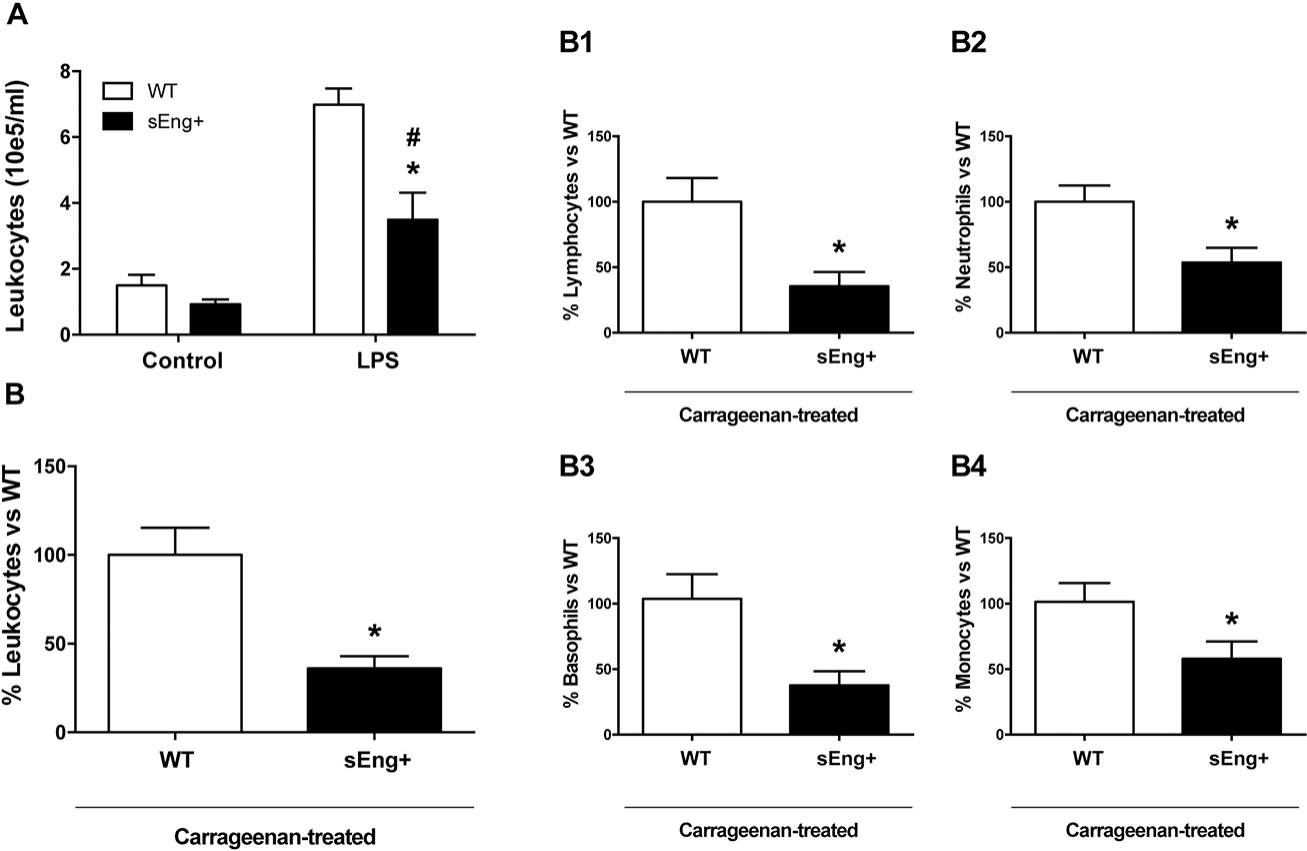
Leukocyte recruitment. (A) Leukocyte recruitment from control and LPS-treated WT and sEng+ mice. Total leukocyte count was measured in BAL. Data are expressed as mean ± SEM. n = 6 in each group of mice, #p<0,005 *vs* control sEng+, T test; *p<0,005 *vs* LPS WT, T test. (B) Leukocyte recruitment in the air pouch lavage from carrageenan-treated WT and sEng+ mice. Data are expressed as mean ± SEM. n = 20 in each group of mice. *p<0,001 *vs* carrageenan WT, T test. (B1) Subpopulations of recruited lymphocytes in air pouch lavage (64,38% inhibition, *p<0,005 *vs* carrageenan WT, T test); (B2) Neutrophils (46,3% inhibition, *p<0,01 *vs* carrageenan WT, T test); (B3) Basophils (66,04% inhibition, *p<0,005 *vs* carrageenan WT, T test); (B4) Monocytes (43,5% inhibition, *p<0,05 *vs* carrageenan WT, T test). n = 20 in each group of mice. Data are expressed as the percentage of sEng+ leukocytes with respect to the WT, mean ± SEM.

Inflammatory cell number in the BAL from WT and sEng+ mice increased significantly 48 h after LPS nebulization compared to the control group, without significant differences between WT and sEng+ control mice. Notably, it was found that soluble endoglin significantly reduced leukocyte concentration in the BAL from LPS treated mice respect to the WT mice (Fig 5A).

The carrageenan model is an established model to study cellular recruitment in response to inflammation which has been widely used in research for its ability to induce an acute inflammatory response. Thus, carrageenan-induced inflammation in the air-pouch model [26] was investigated. In this study, the injection of sterile saline into the pouch produced no inflammatory exudate and no cellular accumulation (leukocyte counts less than 7,0 x 10^5^ i.e. below the sensitivity of the hematology analyzer). In agreement with the above findings, air pouch lavage from sEng+ mice, 24 h after carrageenan injection, contained a smaller number of infiltrated leukocytes than WT mice (Fig 5B). Soluble endoglin significantly reduced total accumulation of cells (63,9% inhibition), as well as lymphocytes (64,38% inhibition) (Fig 5B1), neutrophils (46,3% inhibition) (Fig 5B2), basophils (66,04% inhibition) (Fig 5B3) and monocytes (43,5% inhibition) (Fig 5B4).

**Fig 6:**
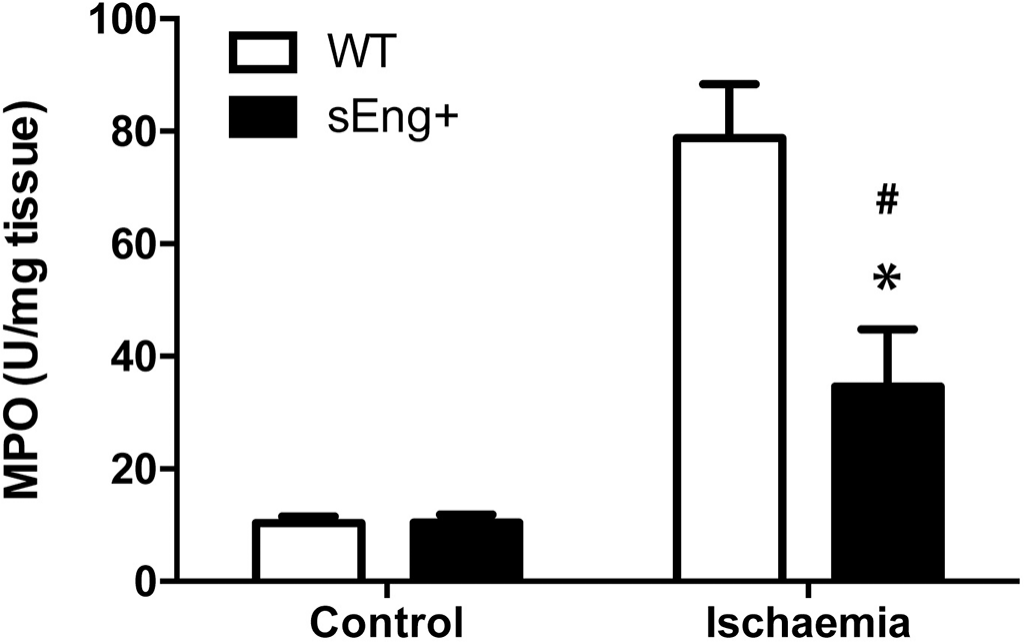
Myeloperoxidase activity. MPO activity in kidneys from control and ischemia-treated WT and sEng+ mice. MPO concentration was measured in kidney tissue and presented as MPO units per milligram of tissue. n = 6 in each group of mice, *p<0,05 *vs* ischemic WT, #p<0,05 *vs* control sEng+, T test.

Myeloperoxidase (MPO) is stored in azurophilic granules of polymorphonuclear neutrophils and macrophages, and is released into extracellular fluid in the setting of an inflammatory process. The MPO activity in control and ischemic kidneys from WT and sEng+ mice was determined. It was observed that MPO activity ischemic kidneys increased significantly 48 h after ischemia-reperfusion compared to the control group. Remarkably, soluble endoglin significantly reduced the increase in MPO activity in ischemic kidneys (Fig 6). No significant differences were found between WT and sEng+ mice under the control conditions.

### Soluble endoglin reduced proinflammatory cytokines levels

**Fig 7:**
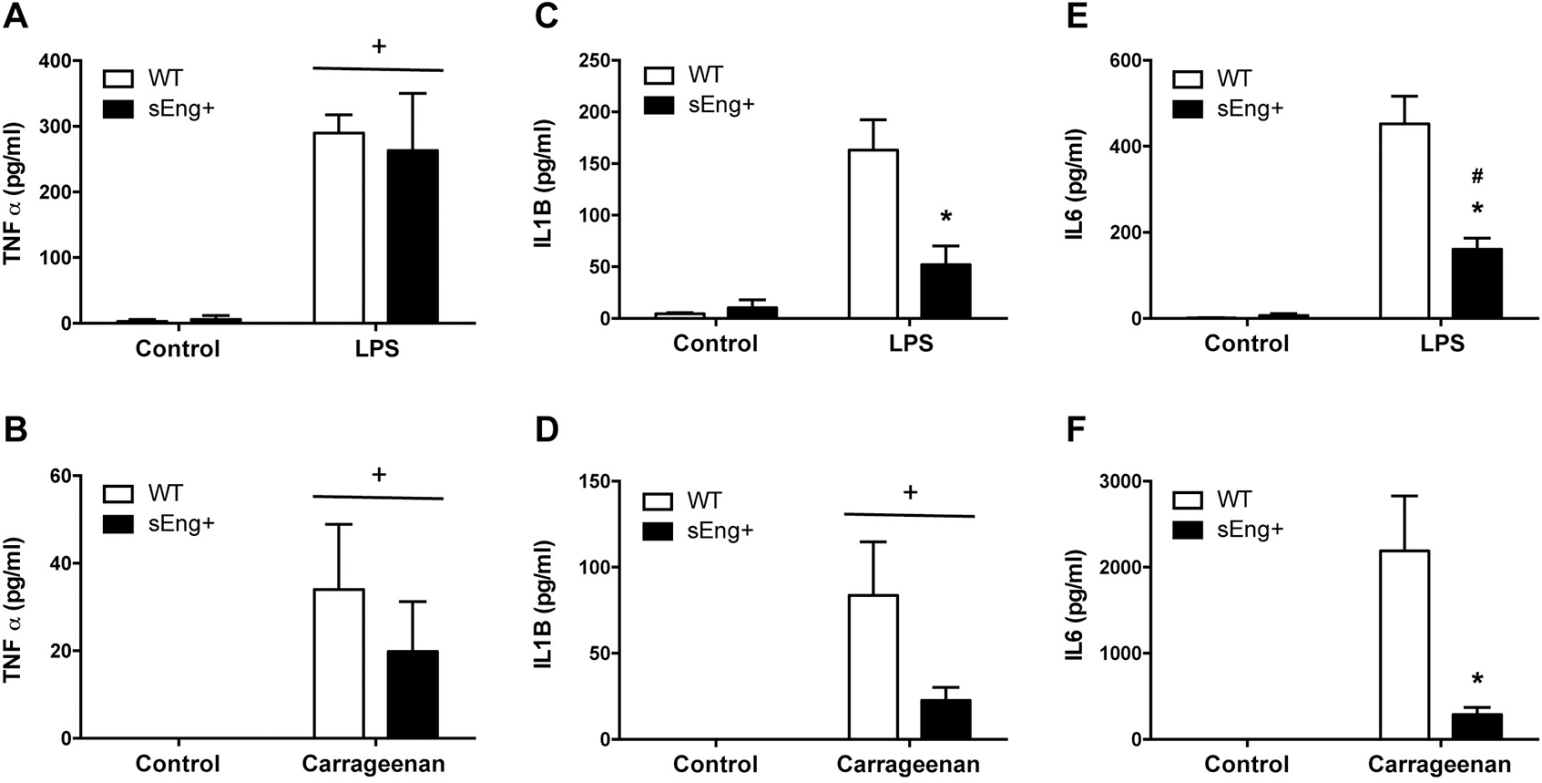
Inflammatory cytokines in BAL and air-pouch lavage. Quantitative analysis of proinflammatory cytokines (TNFα, IL1β and IL6) in BAL and air pouch lavage was performed by ELISA and presented as picograms per milliliter of lavage. Data are expressed as mean ± SEM. n = 5 in each group of mice. (A) TNFα concentration in BAL, +p<0,0001 LPS *vs* control, two-way ANOVA; (B) TNFα concentration in air pouch lavage, +p<0,05 carrageenan *vs* control, two-way ANOVA; (C) IL1β concentration in BAL, *p<0,05 *vs* LPS WT, T test; (D) IL1β concentration in air pouch lavage, +p<0,01 carrageenan *vs* control, two-way ANOVA; (E) IL6 concentration in BAL, #p<0,001 *vs* control sEng+, T test; *p<0,05 *vs* LPS, T test; (F) IL6 concentration in air pouch lavage, *p<0,05 *vs* carrageenan WT, T test.

Inflammatory cytokines have been reported to be involved in neutrophil recruitment and propagation of the inflammatory response. To further evaluate the reduction in inflammation induced by soluble endoglin, levels of proinflammatory cytokines were measured in BAL and air pouch lavage collected from WT and sEng+ mice. It was found that proinflammatory cytokines (TNFα, IL1β and IL6) significantly increased in BAL and air pouch lavage in response to LPS and carrageenan treatment respectively, compared to the control mice receiving saline (Fig 7). Soluble endoglin significantly reduced IL1β and IL6 levels in LPS-treated mice (Fig 7C–7F). However, soluble endoglin did not affect significantly TNFα and IL1β levels in air pouch lavage, although it is observed a reduction tendency (Fig 7B and 7D). Moreover, no significant differences were found between WT and sEng+ mice under control conditions.

**Fig 8:**
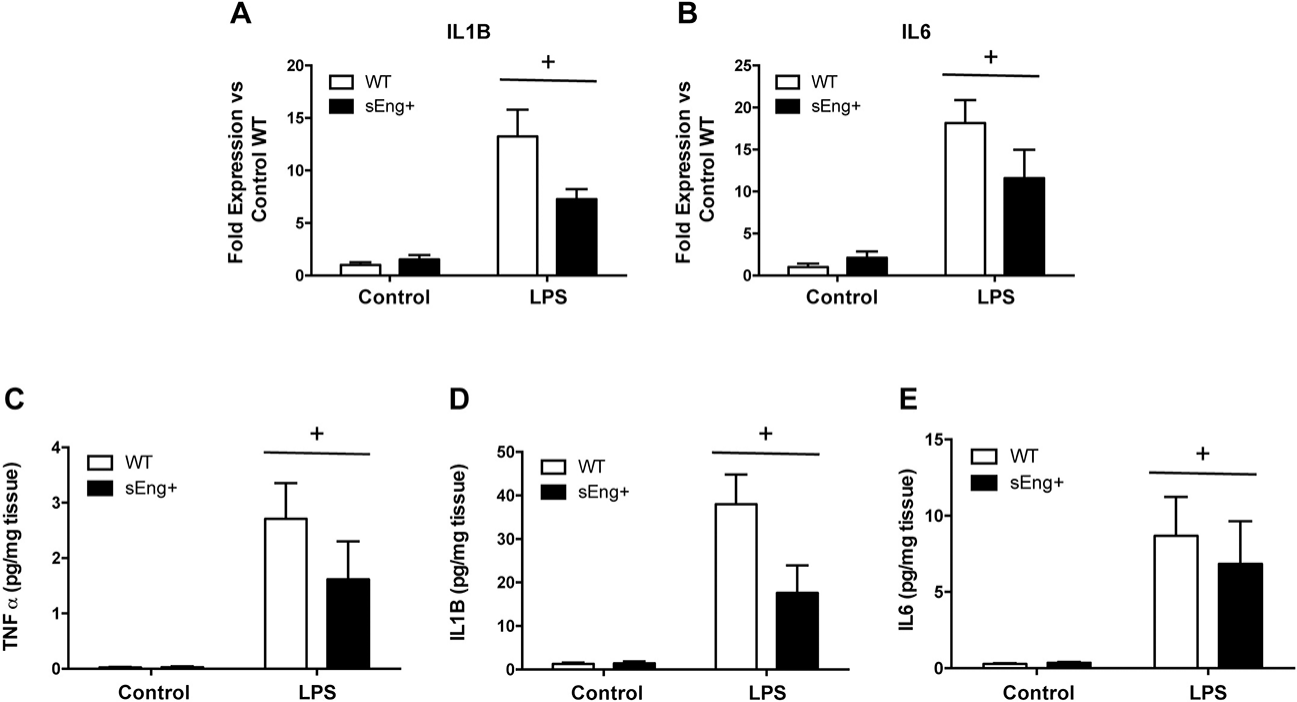
Inflammatory cytokines in lung tissue. Quantitative analysis of inflammatory cytokines (TNFα, IL1β and IL6) in lung tissue was performed by RT-PCR (A-B) and ELISA (C-E). Data are expressed as mean ± SEM. n = 5 in each group of mice. (A) IL1β, +p<0,0005 LPS *vs* control, two-way ANOVA; (B) IL6, +p<0,0001 LPS *vs* control, two-way ANOVA; (C) TNFα, +p<0,005 LPS *vs* control, two-way ANOVA; (D) IL1β, +p<0,005 LPS *vs* control, two-way ANOVA; (E) IL6, +p<0,05 LPS *vs* control, two-way ANOVA.

The expression and presence of proinflammatory cytokines in lungs from WT and sEng+ mice were analyzed using Real-Time PCR and ELISA, respectively. It was found that IL1β and IL6 expression was significantly increased in lungs after LPS treatment. Soluble endoglin effectively decreased the levels of IL1β and IL6 expression compared with the WT LPS group (Fig 8A and 8B), but was statistically significant. TNFα, IL1β, and IL6 protein levels were significantly increased after the LPS inflammatory challenge in WT and sEng+ mice. It was observed that soluble endoglin reduced the increment in TNFα and IL1β (Fig 8C and 8D), but again the result was not statistically significant. No significant differences were found between WT and sEng+ mice under control conditions.

**Fig 9:**
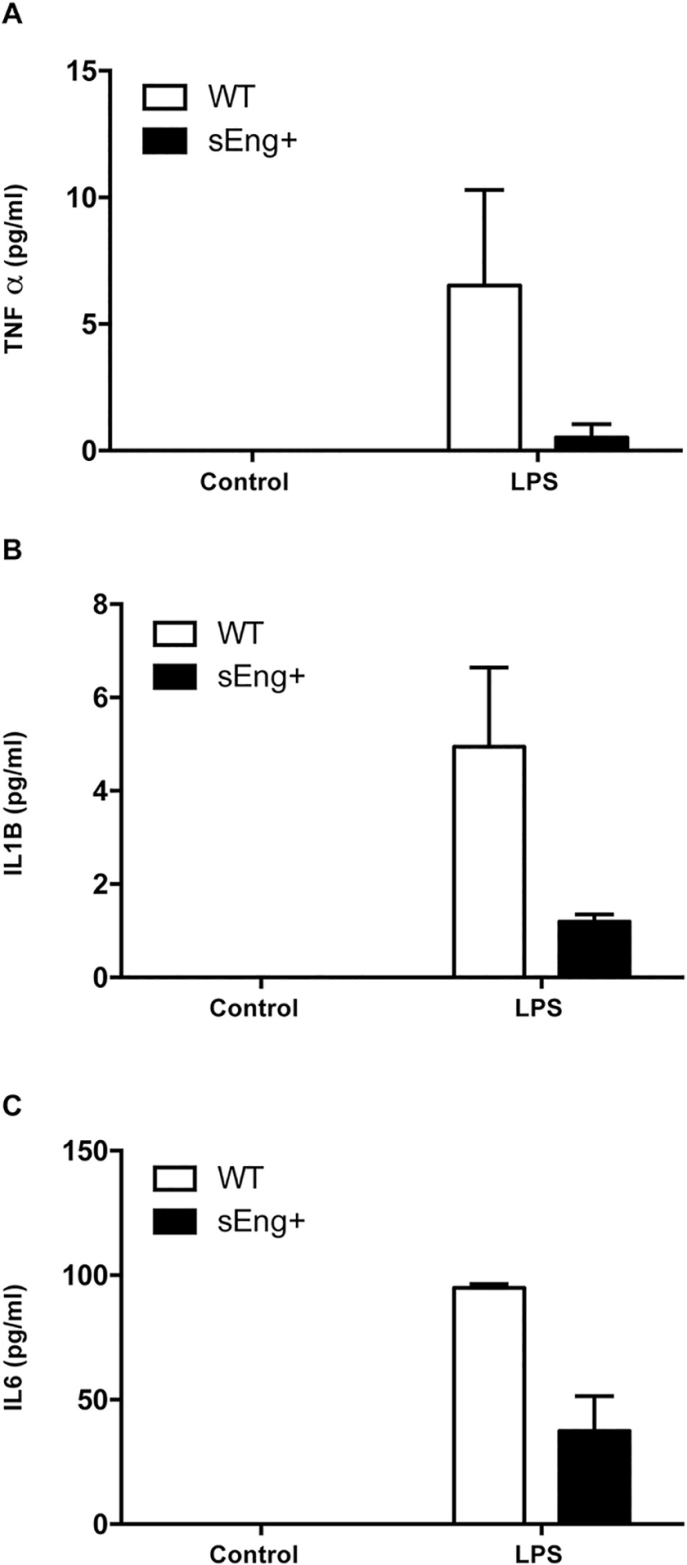
Inflammatory cytokines in plasma. Quantitative analysis of inflammatory cytokines (TNFα, IL1β and IL6) in plasma was performed by ELISA. Data are expressed as mean ± SEM. n = 5 in each group of mice.

The analysis of proinflammatory cytokine levels in plasma is shown in the Fig 9. I was found that the concentration of TNFα, IL1β and IL6 were markedly increased in plasma after LPS challenge. Soluble endoglin did not affect significantly the increase of TNFα, IL1β and IL6 levels in plasma compared with the WT LPS group, although it is observed a reduction tendency in (Fig 9).

### Soluble endoglin reduced inflammation-induced vascular permeability

**Fig 10:**
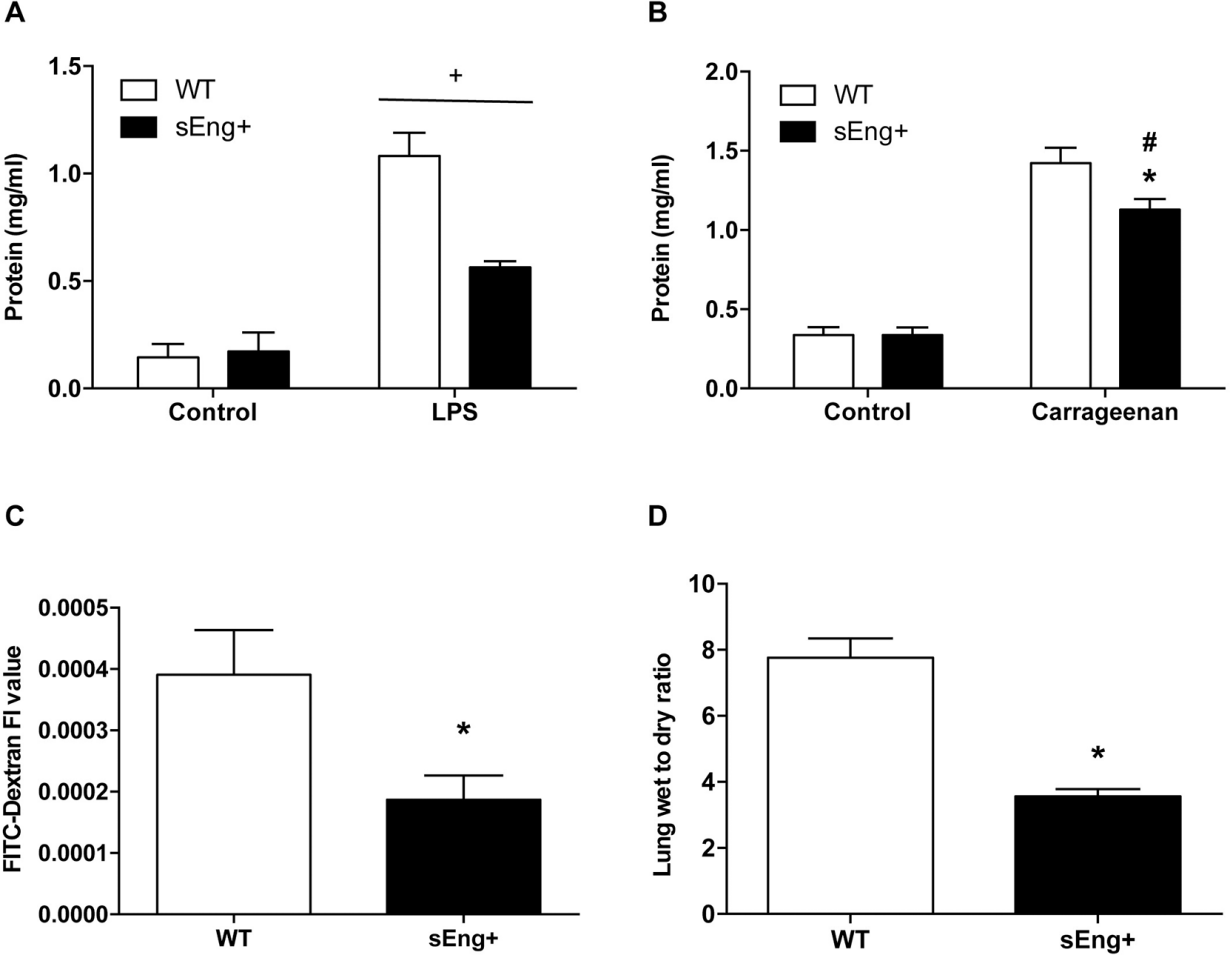
Vascular permeability. (A) Protein concentration in BAL. Data are expressed as mean ± SEM. n = 5 in each group of mice. +p<0,0001 LPS *vs* control, two-way ANOVA; (B) Protein concentration in air pouch lavage. Data are expressed as mean ± SEM. n = 5 in control mice and n = 20 in carrageenan-treated. #p<0,0001 *vs* control sEng+, T test; *p<0,01 *vs* carrageenan WT, T test; (C) Fluorescence in BAL from LPS-treated WT and sEng+ mice. 24 h after LPS exposure, 100 μl of 40kDa FITC-Dextran (25mg/ml) was injected into the retroorbital venous sinus, 30 min before the mice were euthanized. BAL was collected. Data are expressed as mean ± SEM. n = 5 in each group of mice. *p<0,05, T test; (D) Lung wet/dry weight ratios. Data are expressed as mean ± SEM. n = 5 in each group of mice. *p<0,001, T test.

The enhanced recruitment of polymorphonuclear cells in inflamed tissues is associated with an increase in vascular permeability [27]. To evaluate the integrity of the capillary membrane barrier and assess vascular leakage, the protein concentration in BAL and the air pouch lavage, collected from WT and sEng+ mice, was determined. As shown in Fig 10A, the protein levels in BAL were significantly increased in LPS-treated mice compared to the control mice. By contrast, soluble endoglin significantly reduced the increase in protein concentration in LPS-treated mice. No significant differences between WT and sEng+ mice were found in control conditions. Supporting these results, we also found that protein extravasation in the air pouch lavage increased in carrageenan-treated animals (Fig 10B), while soluble endoglin significantly reduced the increase in protein concentration in carrageenan-treated mice. No significant differences between WT and sEng+ mice were found under the control conditions.

To further analyze vascular permeability, the fluorescence in BAL from LPS-treated WT and sEng+ mice after FITC-Dextran intravenous injection was determined. Fig 10C shows the significant reduction of fluorescence induced by soluble endoglin with respect to WT mice. Also, pulmonary edema was examined by measuring the lung wet/dry weight ratio. Fig 10D shows a significant decrease in the wet/dry weight ratio of sEng+ mice compared to the WT LPS-aerosolized mice.

**Fig 11:**
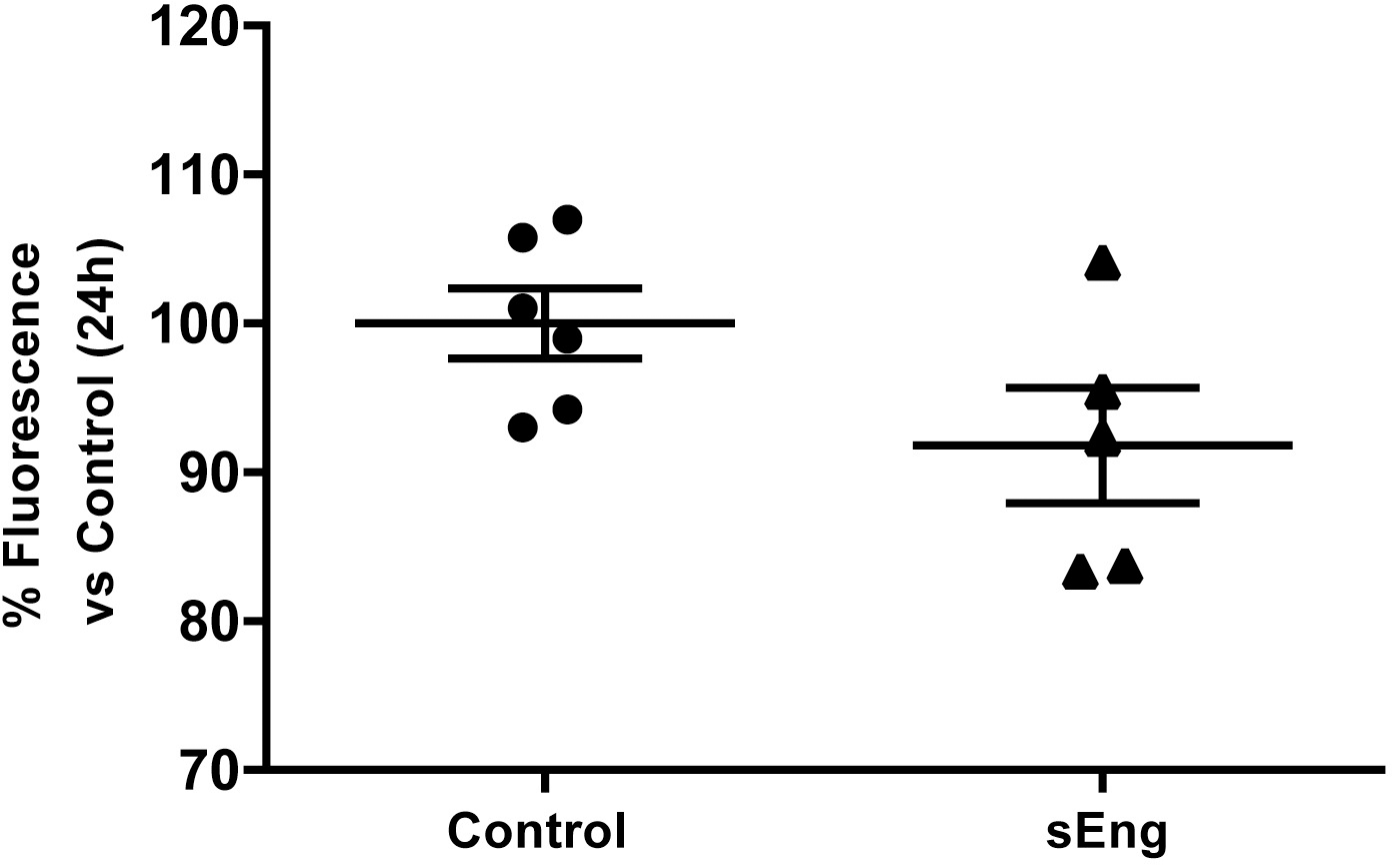
Effect of circulating soluble endoglin on cell permeability. The permeability was determined in endothelial EA.hy926 cells cultured in transwell plates until they reached confluence. Upper chamber media, containing LPS (1 μg/ml) and soluble endoglin (500 ng/ml) for their respective treatments, were replaced with FITC-Dextran (40 kDa) at 1 mg/ml in DMEM. After 24 h at 37°C, the inserts were removed, and the amount of fluorescence in the bottom chambers was measured using a fluorescence plate reader. Data are presented as percentage of fluorescence versus Control. Data are expressed as mean ± SEM. n = 6 in each group of mice. T test, p = 0,09.

In addition, i*n vitro* experiments showed that soluble endoglin decreased the permeability to FITC-Dextran of a LPS-activated human endothelial cell EA.hy926 (Fig 11).

**Fig 12:**
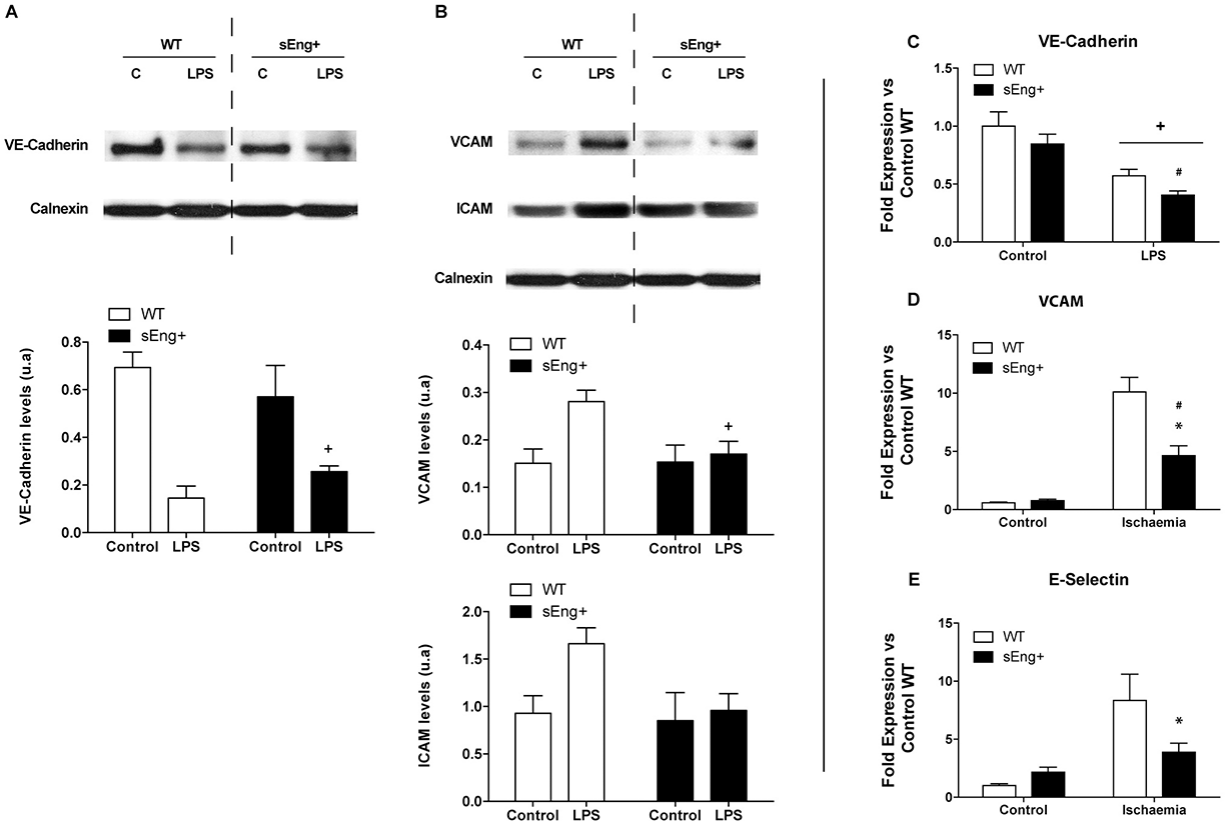
Effect of circulating soluble endoglin on endothelial adhesion molecules. (A) VE-Cadherin amount of protein and mRNA expression were determined by western blot: +p<0,0001 LPS *vs* control, two-way ANOVA; and RT-PCR (C): +p<0,0001 LPS *vs* control, #p<0,05 *vs* control sEng+; two-way ANOVA. (B) VCAM and ICAM amount of protein was determined by western blot, +p<0,05 LPS *vs* control, two-way ANOVA. (D)VCAM mRNA expression was also determined by RT-PCR in kidney tissue: *p<0,01 *vs* ischemic WT, #p<0,005 *vs* control sEng+, T test. Equal loading of samples was confirmed by immunodetection of calnexin. Top: Representative immunoblots. Bottom: densitometric analysis. Data are expressed as mean ± SEM. n = 5 in each group of mice. (E) E-Selectin mRNA expression was determined by RT-PCR. E-Selectin, *p<0,05 *vs* ischemia WT, T test.

Endothelial permeability is regulated in part by the dynamic opening and closure of cell-cell adherent junctions. In endothelial cells, adherent junctions are largely composed of vascular endothelial cadherin (VE-cadherin), an endothelium-specific member of the cadherin family of adhesion proteins that binds, via its cytoplasmic domain, to several protein partners [28]. To evaluate the role of soluble endoglin in the capillary membrane barrier, VE-cadherin amount of protein in lung tissue from WT and sEng+ mice was determined. The amount of VE-cadherin was lower in LPS-treated mice compared to the control mice. Soluble endoglin did not affect significantly the amount of VE-cadherin in LPS-treated mice, although it is observed a reduction tendency (Fig 12A). Furthermore, it was found that VE-Cadherin amount of protein was significantly lower in lungs after LPS challenge (Fig 12C). No significant differences were found between WT and sEng+ mice under control conditions.

### Soluble endoglin modified inflammation-induced endothelial adhesion molecules expression

During inflammation, local innate cells release cytokines and vasoactive compounds that induce activation of endothelial cells by increasing permeability and upregulating adhesion molecules expression (i.e. Intercellular adhesion molecule (ICAM-1) and vascular cell adhesion molecule (VCAM-1). The results of western blotting indicated that LPS treatment resulted in a significant induction of ICAM-1, VCAM-1 in lung tissues. Soluble endoglin decreased this adhesion molecules amount of protein in LPS-treated mice (Fig 12B). VCAM and E-selectin mRNA expression were significantly increased in kidneys after ischemia-reperfusion. Soluble endoglin significantly decreased the levels of VCAM and E-selectin mRNA expression compared to the WT ischemia group (Fig 12D and 12E). No significant differences between WT and sEng+ mice under the control conditions were found.

## Discussion

There is a growing body of research supporting the role of inflammation in cardiovascular diseases. Hypertension is the most common chronic disease and the major cause of heart failure, stroke, chronic kidney disease and mortality. Experimental and clinical evidence suggests inflammation has a critical role in the development of hypertension [29,30]. Inflammation is one of the main reasons why people with diabetes experience heart attacks, strokes, kidney pathologies and other related complications [31–34]. Most diabetic treatments also exert anti-inflammatory effects [32]. Atherosclerosis also has been recognized as an inflammatory disease of the arterial wall [35]. Furthermore, preeclampsia, a serious condition that affects 5–10% of all pregnancies [36], characterized by hypertension and proteinuria [37] and other systemic disturbances, [38] is also associated with a systemic inflammatory response [39–42]. In addition, circulating levels of IL6, tumor necrosis factor alpha (TNFα) and monocyte chemoattractant protein 1 (MCP-1) have been found to be elevated in preeclampsia [43].

Interestingly, the above mentioned diseases exhibit elevated levels of circulating soluble endoglin, including hypercholesterolemia [44], hypertension [16], atherosclerosis [45], type II diabetes mellitus [8] and preeclampsia [46]. Soluble endoglin has been shown to be a potential biomarker of these diseases [47], however, the direct relationship between the elevated levels of soluble endoglin and the inflammatory processes involved in these diseases still remains to be elucidated.

The sEng+ mice used in the present study have elevated plasma levels of soluble human endoglin and develop mild hypertension and proteinuria as it has been published [16]. Inflammation induce an increase in the amount of mouse membrane endoglin protein after inflammation in lung and kidney tissues without significant differences between sEng+ and WT mice. Recently, it has been shown, in an *in vitro* study, that soluble endoglin induce an increase in the levels of membrane endoglin [48]. However, the effects of soluble endoglin on endothelial cells in culture are not demonstrated to be mediated by membrane endoglin. Accordingly, our work supports the hypothesis that soluble endoglin has a role itself rather than exerts its function by antagonizing the role of membrane endoglin. Some studies have postulated that the effect of soluble endoglin is produced by antagonizing the effect of membrane endoglin or by sequestering the ligand. Soluble endoglin can bind several ligands, among them TGF-β1, BMP-9 and BMP-10 [5,14]. When soluble endoglin binds circulating TGF-β1, the availability of this cytokine to interact with its membrane receptors decreases, as soluble endoglin is unable to interact directly with the extracellular region of TGF-β receptors type I and type II [15]. Further studies are needed to elucidate the relationship between the effects of soluble endoglin and membrane endoglin.

In this study, we show that high levels of soluble endoglin alone do not modify the inflammatory state of sEng+ mice. Our results show no differences in the inflammatory state (leukocyte infiltration, inflammatory cytokines, vascular permeability or adhesion molecules expression) between WT and sEng+ mice under control conditions. In agreement to this finding, Nemeckova et al., also show that high levels of soluble endoglin alone do not induce differences in leukocyte recruitment, inflammatory cytokines, vascular permeability or adhesion molecules expression in the tissues of both sEng+ and control mice [44].

In addition, it has been shown that soluble endoglin does not induce endothelial dysfunction under control conditions *in vivo* [44,49]. Recently, Varejckova et al. found, in an *in vitro* study, that soluble endoglin treatment results in an activation of NF-κB/IL6 expression, without significant effects on other markers of endothelial dysfunction and inflammation, including eNOS, peNOSS1177, VCAM-1, COX-1, COX-2 and ICAM-1 [48]. The fact that other NF-κB-regulated pro-inflammatory proteins were not affected by the addition of soluble endoglin suggests that soluble endoglin treatment by itself does not induce an inflammatory response in endothelial cells in this experimental design.

Our results do not rule out the possibility that soluble endoglin may contribute to the alteration of the inflammatory state when accompanied by a second hit, similar to what occurs in inflammatory diseases. To this purpose, an animal model was employed where increasing circulating levels of soluble endoglin is matched with an inflammatory process. The animals experienced acute inflammation using three approaches: LPS nebulization, carrageenan air pouch and renal ischemia-reperfusion. Systemic inflammation is rapidly activated by LPS administration in experimental models, which subsequently increases the plasma levels of inflammatory cytokines (TNFα or IL1β) and oxygen-free radicals [50,51].

In the present study, LPS nebulization induces tissue damage, characterized by increased lung water content, disruption of lung architecture, extravasation of red blood cells and accumulation of inflammatory cells. These results are consistent with the observations of other authors in LPS-treated animals [52–54]. Ischemia-reperfusion injury (IRI) is characterized by restriction of blood supply to an organ followed by restoration of blood flow and re-oxygenation. This phenomenon exacerbates tissue damage by initiating an inflammatory cascade including reactive oxygen species, cytokines, chemokines, and leukocytes activation [55–57].

The lung and kidney injury induced by the inflammatory agents was reduced in sEng+ mice, especially the intra-alveolar and kidney infiltrates, suggesting a possible decrease of inflammation induced by soluble endoglin. To deepen into this effect, the leukocyte number in BAL and air pouch lavage was evaluated. Our results demonstrate that soluble endoglin reduced the recruitment and transmigration of leukocyte in this mice model. In addition, we observed a significant reduction of neutrophil infiltration in LPS-treated lungs and ischemic kidneys from sEng+ with respect to WT mice. Supporting this observation, Rossi et al., showed that soluble endoglin inhibits leukocyte adhesion and transmigration *in vitro* [58]. They also demonstrated that leukocyte transmigration was lower in endoglin deficient mice and that the RGD endothelial endoglin motif interacts with the leukocyte integrin α5β1, suggesting a regulatory role for membrane endoglin in transendothelial leukocyte trafficking. The interaction between soluble endoglin and leukocyte integrins could explains our results regarding leukocyte transmigration, however an integrin-independent effect of soluble endoglin should not be discarded. Since soluble endoglin could bound the leukocytes integrins and prevent the transmigration through the endothelial cells, although the permeability and/or the proinflammatory cytokines have to be considered. In addition, soluble endoglin contains the binding site for different members of the TGF-β superfamily and may act as a scavenger of circulating ligands, preventing their binding to the functional receptors [5,9]. However, the possible role of soluble endoglin antagonizing the effect of membrane endoglin is a hypothesis that is not proven yet. Our results demonstrate the involvement of soluble endoglin in the regulation of permeability and proinflammatory cytokines. These are important and novelty mechanisms of the inflammatory process regulation that here demonstrate to be regulated by soluble endoglin. Additionally, the mechanisms through which soluble endoglin prevents inflammation were studied. It is well known that LPS and carrageenan stimulate macrophages/monocytes to sequentially release proinflammatory cytokines and that TNFα and IL6 participate in the early development of inflammation [59,60]. In the current study, we found that LPS significantly increases BAL and tissue proinflammatory cytokines TNFα, IL1β and IL6, whereas soluble endoglin prevents this increase.

Jezkova et al. found that in female, but not in male mice, proinflammatory markers were higher in the aortas of sEng+ mice 3 months after the intake of high-fat diet [61]. Our study differs substantially from that of Jezkova et al. because, we induce an acute inflammation under the effect of circulating soluble endoglin. While in the Jezkova study inflammatory markers are studied 3 months after the insult, there may be other processes involved in the analyzed response. It also should be considered that differences in inflammatory markers attributed to soluble endoglin appear only in female mice, which means that the mechanisms involved in the process could be related with sex factors.

Modifications in the inflammatory process may explain damage to the vascular endothelium which leads to the capillary leak [62–64]. In this study, we clearly demonstrate that inflammation-induced vascular permeability is reduced by soluble endoglin. To our knowledge, this is the first time that the relationship between circulating soluble endoglin and inflammation-induced vascular permeability has been shown. The formation of new immature and leaky vessels along with inflammatory remodeling accompany the development of numerous diseases beyond cancer, and present an opportunity for passive accumulation of intravenously administered nanomedicines in many pathological tissues [65]. Soluble endoglin could represent an opportunity and create new therapy and prevention technologies for many disorders. For example, therapeutic strategies to limit inflammatory cells and their products have been successful in pre-clinical tumor models [66]. Furthermore, in the systemic pregnancy syndrome of preeclampsia, both, systemic inflammation and soluble endoglin, have been found elevated [67]. However, the fact that both parameters are elevated in preeclampsia does not demonstrate that soluble endoglin induce inflammation. We do not know how the inflammation in preeclampsia would be in the absence of soluble endoglin, it might be that we would found a greater inflammation than that observed with elevated levels of circulating endoglin. Preeclampsia is a complex process that evolves throughout pregnancy. Effectively soluble endoglin has been shown to be a molecule involved in the pathogenesis of preeclampsia [16]. Our results do not contradict this affirmation. Numerous studies have shown that soluble endoglin is elevated as early as 11–13 weeks of gestation before the development of preeclampsia [67]. In contrast, neutrophil activation and elevation of Th1 pro-inflammatory cytokines have been shown to occur at the time of diagnosis of preeclampsia [67]. The models of acute inflammation in the presence of circulating endoglin used in this study could allow us to approach a hypothetical onset of preeclampsia, where the presence of high levels of soluble endoglin precedes the inflammatory processes. In this context, the reduction of inflammation induced by circulating soluble endoglin may explain the antiangiogenic effect of soluble endoglin in the early stages of preeclampsia. Hence, the initiation of angiogenesis is often associated with an increased capillary permeability that serves to enrich the adjacent interstitial compartment with plasma components. Inflammation and angiogenesis are thereby linked processes, but exactly how they are related has not been well understood [68].

This study also shows that soluble endoglin prevents the increased expression of adhesion molecules, ICAM, VCAM and E-selectin induced by the inflammatory stimulus. ICAM, VCAM and E-selectin are essential for stable adhesion and transmigration of leukocytes in most types of inflammatory processes and blocking antibodies against ICAM-1 inhibits leukocyte adhesion. The reduction in the adhesion molecules expression can lead to a decrease in the number of transmigrated leukocytes and thus could explain the decrease of inflammatory cytokines in the presence of soluble endoglin. The inflammation-induced disruption of adherent junctions is associated to the decreased expression of VE-cadherin mRNA. One consequence of adherent junction disassembly is that the compromised endothelial cell barrier lead to an influx of solutes and increased neutrophil infiltration. Therefore, circulating soluble endoglin could prevent the increase in inflammation-induced vascular permeability that occurs during inflammation-related diseases, preventing the increase of inflammatory cytokines due to the reduction of inflammatory cells recruitment.

This work brings out an interesting relationship between circulating soluble endoglin and diseases with an inflammatory component. When elevated levels of soluble endoglin are accompanied by an inflammatory process, the result is a decrease in vascular permeability, a reduction in the proinflammatory biomarkers TNFα, IL1β and IL6, and less leukocyte recruitment and leukocyte tissue infiltration. Taken together, these results, indicate that circulating soluble endoglin may contribute to a lesser increase in the inflammatory response.

These encouraging results represent a turning point in the understanding of the role of soluble endoglin in the pathophysiology of various cardiovascular diseases, and, imply that soluble endoglin has an impact on the inflammatory state during inflammatory-related diseases. These results provide a better understanding of the inflammatory basis of these diseases, and open new perspectives leading to the development of novel and targeted approaches for the prevention and treatment of cardiovascular disease.

## Supporting information

S1 Fig Gel images used to make the figures. (PDF)

S2 Fig ARRIVE guidelines checklist. (PDF)
