# Supplementary material for: Circulating soluble endoglin modifies the inflammatory response in mice
Source: PLoS One. 2017 Nov 16;12(11):e0188204. doi: 10.1371/journal.pone.0188204 (PMC5690682; doi:10.1371/journal.pone.0188204)

Gel images used to make the figures

Fig 2. Membrane endoglin (lung) &Calnexin

Membrane endoglin (lung)

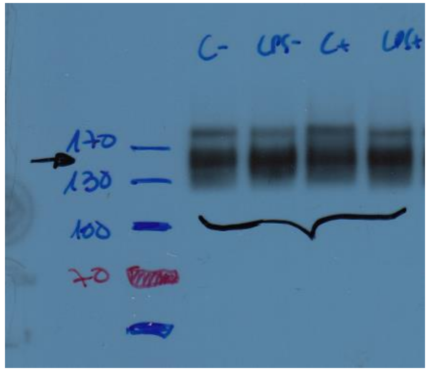

Calnexin

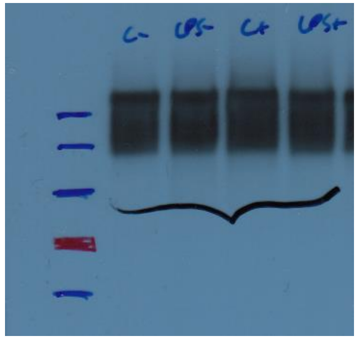

Fig 2. Membrane endoglin (kidney) &Calnexin

Membrane endoglin (kidney)

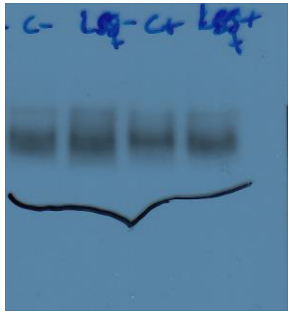

Calnexin

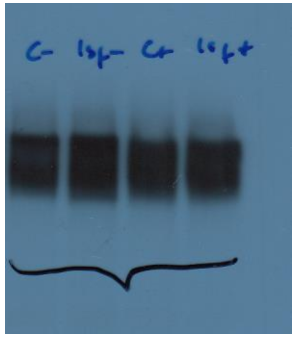

Fig 12. VCAM-1(lung) &Calnexin

VCAM-1(lung)

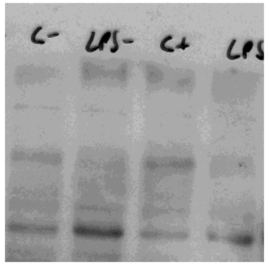

Calnexin

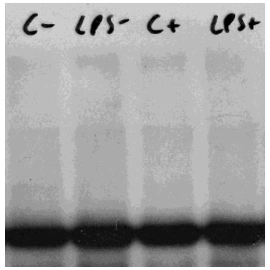

Fig 12. ICAM-1(lung) &Calnexin

ICAM-1(lung)

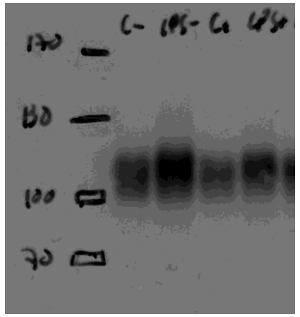

Calnexin

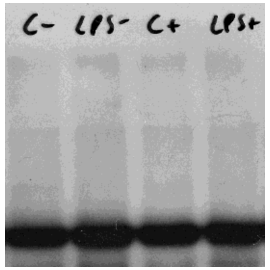

Fig 12. VE-Cadherin(lung) &Calnexin

VE-Cadherin (lung)

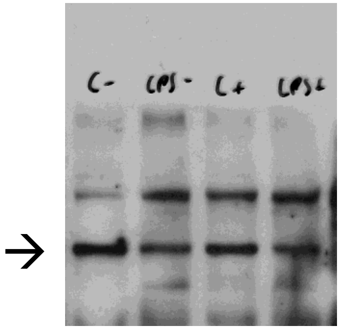

Calnexin

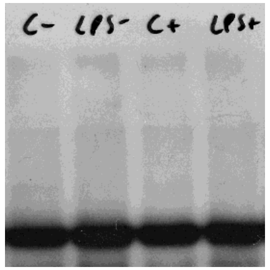

Supplement: S1 Fig — (PDF) [file pone.0188204.s001.pdf]
